# Adolescents’ Involvement in Cyber Bullying and Perceptions of School: The Importance of Perceived Peer Acceptance for Female Adolescents

**DOI:** 10.1007/s11199-017-0742-2

**Published:** 2017-03-15

**Authors:** Lucy R. Betts, Karin A. Spenser, Sarah E. Gardner

**Affiliations:** Department of Psychology, Nottingham Trent University, 50 Shakespeare Street, Nottingham, NG1 4FQ UK

**Keywords:** Cyber bullying, Spillover, Value of learning, Perceptions of peers, Victim, Bully, Bully/victim, School adjustment, Trust, Self-esteem, Psychosocial adjustment, Attitudes towards school

## Abstract

Young people are spending increasing amounts of time using digital technology and, as such, are at great risk of being involved in cyber bullying as a victim, bully, or bully/victim. Despite cyber bullying typically occurring outside the school environment, the impact of being involved in cyber bullying is likely to spill over to school. Fully 285 11- to 15-year-olds (125 male and 160 female, *M*_age_ = 12.19 years, *SD* = 1.03) completed measures of cyber bullying involvement, self-esteem, trust, perceived peer acceptance, and perceptions of the value of learning and the importance of school. For young women, involvement in cyber bullying as a victim, bully, or bully/victim negatively predicted perceptions of learning and school, and perceived peer acceptance mediated this relationship. The results indicated that involvement in cyber bullying negatively predicted perceived peer acceptance which, in turn, positively predicted perceptions of learning and school. For young men, fulfilling the bully/victim role negatively predicted perceptions of learning and school. Consequently, for young women in particular, involvement in cyber bullying spills over to impact perceptions of learning. The findings of the current study highlight how stressors external to the school environment can adversely impact young women’s perceptions of school and also have implications for the development of interventions designed to ameliorate the effects of cyber bullying.

Young people’s experiences of bullying are evolving. Previously bullying experiences were typically confined to school and would end with the school day. However, our increasing connectivity, the rapidly evolving digital world, and the pervasiveness of technology have together transformed face-to-face bullying in to a new form: cyber bullying (Huang and Chou 2010). A recent meta-analysis proposed that cyber bullying involves four distinct components: “(a) intentional aggressive behaviour that is, (b) carried out repeatedly, (c) occurs between a perpetrator and victim who are unequal in power, and (d) occurs through electronic technologies.” (Kowalski et al. 2014, p. 1073). Although the reported prevalence rates of involvement in cyber bullying converge between 20 and 40% (e.g., Dehue et al. 2008), some studies report far greater involvement in cyber bullying. For example, Juvonen and Gross (2008) stated that 72% of their sample reported that they were a victim of cyber bullying, Xiao and Wong (2013) identified that 60% of their sample reported that they engaged in behaviours consistent with cyber bullying, and Brack and Caltabiano (2014) reported that 62% of their sample were bully/victims who engage in cyber bullying behaviours and experience them.

The consequences of involvement in face-to-face bullying are well documented for psychosocial adjustment (e.g., Olweus 2013; Smith 2004) and perceptions of school (e.g., Gruber and Fineran 2016); however, comparably fewer studies have explored the consequences of involvement in cyber bullying. Moreover, one of the defining features of cyber bullying is that it can occur at any time (Snakenborg et al. 2011) and, as such, researchers have argued that the potential impact of cyber bullying is greater than face-to-face bullying (Nixon 2014). Further, given the potential 24-h nature of cyber bullying, it is likely that experiences of cyber bullying and the associated stress and negative affect spillover into other aspects of young people’s lives. Therefore, the current study examined the impact of 11- to 15-year-olds’ self-reported involvement in cyber bullying as a victim, bully, or bully/victim on their perceptions of learning and the value of school. Involvement in cyber bullying was operationalised through separate self-reports of (a) experiencing cyber bullying behaviours akin to being a victim and (b) engaging in cyber bullying behaviours akin to being a bully. The self-reports for experiences as a victim and engaging in cyber bullying were also combined to identify bully/victims.

## Cyber Bullying and School Adjustment

Although cyber bullying typically occurs outside the school environment, up to a third of young people report that cyber bullying affects them at school (Patchin and Hinduja 2006). One theoretical explanation for this pattern of results resides in the spillover that occurs between home and school such that when a young person experiences stressors in one of these environments, the effects are evidenced in the other (Timmons and Margolin 2015). There is some empirical evidence of such spillover between cyber bullying experiences and the school environment. For example, many young people who experience cyber bullying as a victim report that they are afraid to go to school (Raskauskas and Stoltz 2007) and, for some young people, this fear escalates to active avoidance manifested as truancy (West 2015; Ybarra et al. 2007). Involvement in cyber bullying also results in young people feeling less safe in school (Sourander et al. 2010) and having negative attitudes towards school (Bayar and Ucanok 2012; Pyzalski 2012). However, in a cross-sectional study of seventh grade students, Li (2007) reported that half of those young people who were victims of cyber bullying had above average school grades. Most of the previous research has tended to focus exclusively on the impact of cyber bullying on school adjustment either from the perspective of the victim (e.g., Sourander et al. 2010) or the bully (e.g., Pyzalski 2012); however, there is growing evidence that many young people fulfil both the bully and victim role simultaneously in cyber bullying (Lam et al. 2013). Therefore, in the current study we simultaneously examined young people’s experiences as the victim, bully, and bully/victim in cyber bullying, and we expected that involvement in cyber bullying (as a victim, bully, or bully/victim) will be associated with negative perceptions of learning and school (Hypothesis 1).

## Mechanisms of Influence

There are three possible mechanisms through which involvement in cyber bullying may influence young people’s perceptions of learning and school: (a) self-esteem, (b) trust, and (c) perceived peer acceptance. Together, self-esteem, trust, and perceived peer acceptance previously have been found to be influenced by involvement in bullying and cyber bullying and impact young people’s school adjustment. Further, these variables underpin social interactions (Rogers 1959). Consequently, the present study examined these variables as potential mediators in the relationship between cyber bullying involvement and perceptions of learning and school. Examining potential mediators in this relationship is appropriate because in previous studies a portion of the variance remains unaccounted for, implicating the role of other variables in the relationship (Barchia and Bussey 2010).

Having higher levels of self-esteem is predictive of indicators of young people’s school adjustment (Alves-Martines et al. 2002) and academic achievement (Hamid et al. 2013). Further, higher self-esteem facilitates young people’s self-regulatory behaviours in an academic context which are an important prerequisite for promoting academic performance (Di Giunta et al. 2013). The relationship between face-to-face bullying and self-esteem has been clearly established in the literature (Hawker and Boulton 2000), and there is emerging evidence of a similar association between cyber bullying and self-esteem (Cénat et al. 2014; Patchin and Hinduja 2010). For example, involvement in cyber bullying either as a victim or bully is associated with lower self-esteem (Didden et al. 2009). Further, there is emerging evidence that self-esteem likely operates as a protective buffer from some of the negative consequences of involvement in cyber bullying such that higher levels of self-esteem go some way to ameliorate the negative consequences of involvement in cyber bullying (Álvarez-García et al. 2015). Therefore, we examined the role of self-esteem as a potential mediator in the relationship between involvement in cyber bullying and perceptions of learning and school.

The propensity to trust others is important for social relationship formation and maintenance (Rotenberg 1994) and school adjustment (Betts and Rotenberg 2007; Betts et al. 2009). Betts et al. (2017) have previously argued that experiencing bullying is likely to influence young people’s cognitive schemas such that they become less trusting and that this in turn negatively impacts adjustment and perceptions of school. Trust may mediate the relationship between involvement in cyber bullying and perceptions of learning and school because involvement in cyber bullying likely impacts an individual’s propensity to trust others and because collaborative learning with peers at school is a frequently implemented technique to facilitate knowledge transfer (Davies et al. 2013). Young people also report that if the true identity of the perpetrator of cyber bullying is unknown, this fosters a greater sense of suspicion and weariness when interacting with others (Raskauskas and Stoltz 2007). Consequently, these perceptions of mistrust arising from involvement in cyber bullying may spill over to influence young people’s social cognitions which, in turn, may lead them to have negative perceptions of learning and school. Consequently, we explored the role of trust as a potential mediator in the relationship between involvement in cyber bullying and perceptions of learning and school.

The extent to which children feel part of the class group and accepted by their peers influences their psychosocial adjustment (Pardini et al. 2006) and their interest in school (Wentzel 1998). Similar to trust, young people’s experiences of cyber bullying may also impact their perceived peer acceptance which may, in turn, influence their perceptions of learning and school. For example, Jackson and Cohen (2012) reported that third to sixth graders who were victims of cyber bullying were more likely to report lower levels of optimism, fewer friendships, and lower social acceptance than those not involved in cyber bullying. Young people who experience cyber bullying also report that they perceive themselves to be less popular (Vandebosch and van Cleemput 2009). In addition to influencing young people’s perceptions of their social acceptance, involvement in cyber bullying as a victim may foster a sense of reluctance to interact in the social world. Conversely, young people who engage in cyber bullying behaviours have higher levels of perceived popularity (Wegge et al. 2016). More generally, there is evidence that young people’s perceptions of the quality of their peer relationships impact their school engagement (Lynch et al. 2013). Consequently, we examined perceived peer acceptance as a potential mediator in the relationship between involvement in cyber bullying and perceptions of learning and school.

The role of students’ gender was also explored as a potential moderator in the relationship between cyber bullying involvement, self-esteem, trust, perceived peer acceptance, and perceptions of learning and school. Previous research has suggested that females are more likely to experience cyber bullying (Dehue et al. 2008; Festl and Quandt 2013; Kowalski and Limber 2007) and report more distress when they experience cyber bullying (Bauman and Newman 2013) whereas other research has reported that males are more likely to experience cyber bullying (Erdur-Baker 2010). The evidence regarding the gender of cyber bullies is similarly mixed; some studies report that males are more likely to be the perpetrators of cyber bullying than females (Gradinger et al. 2009; Lapidot-Lefler and Dolev-Cohen 2015), whereas other studies have reported that females are more likely to be perpetrators (Connell et al. 2014). Young men are also more likely to admit their own wrongdoing in anonymous digital posts about victimization (Thomas et al. 2016).

There are also reported gender differences in young people’s peer relationships. Females are more likely to be more strongly attached to their peers (Gorrese and Ruggieri 2012) and perceive them to be more supportive (Lam et al. 2012) than males and, as such, the consequences of involvement in cyber bullying may be greater for young women’s social relationships. Young women also report higher levels of school engagement and have more positive perceptions of learning than young men do (Lam et al. 2012; Wang and Eccles 2012). Therefore, we predict that gender will moderate the relationship between cyber bullying involvement as a victim, bully, or bully/victim and negative perceptions of learning and school, via self-esteem, trust, and perceived peer-acceptance (Hypothesis 2).

## Method

### Participants

Four secondary schools in the Midlands of the United Kingdom were invited to participate in the research, and one school agreed. Initially, 345 (153 male, 192 female) 11- to 15-year-olds returned the pencil-and-paper questionnaire. Any incomplete questionnaires were removed from the dataset (*n* = 60), yielding a final sample of 285 (125 young men and 160 young women, *M*_age_ = 12.25 years, *SD* = 1.09) and an 82.60% response rate. There was no significant gender difference in completion, χ^2^(1) = .09, *p* = .886, but participants who completed the questionnaires were older (*M* = 12.21, *SD* = 1.04) than those who did not complete the questionnaires (*M* = 11.73, *SD* = .59), *t*(242.84) = 5.37, *p* < .001. The school had a catchment area that served a range of socio-economic areas, and a majority of the sample was White.

### Procedure

Participants completed the questionnaires individually during class time. Before participating in the research, students were told that participation was voluntary, that they were free to stop answering the questions at any time, that there were no correct answers, and that all answers would be confidential (unless they disclosed a significant risk of harm). Consent for the students’ involvement in the research was initially given by the head teacher of the participating school. Letters that outlined the nature of the research were then sent to the parents of the students and, having received this information, parents were asked to contact the school if they did not want their son/daughter to participate in the study. All parents agreed that their son/daughter could participate in the study. The young people were asked to give their assent before completing the measures. The survey included the measures in the order that follows, and it took about 50 min to complete.

The percentage of missing data for items relating to involvement in cyber bullying ranged from 0% to 3.5%. For the mediators (self-esteem, trust, and perceived peer acceptance) between .4% and 3.9% of the items contained missing data. For the outcome variable (perceptions of learning and school) between 0% to 3.9% of items had missing data. The missing data mechanism was assessed using Little’s (1988) MCAR test statistic. For the sample of 285 cases, the missing data were not missing completely at random (*X* ^2^ = 6962.82, df = 6329, *p* < .001). However, although the Little’s MCAR test statistic suggests that data are not MCAR, it is argued that a missing rate of 5% or less is unlikely to produce biased parameter estimates (Cheema 2014; Schafer 1999).

### Measures

#### Cyber Bullying Involvement

Young people’s involvement in cyber bullying over the last 3 months was assessed using the Cyber Victimisation Experiences and Cyber Bullying Behaviours scales (Betts and Spenser 2017). The Cyber Victimisation Experiences scale comprises 15 items across three subscales: threats (6 items, e.g., “Sent me a threatening comment anonymously,” α = .83), sharing images (5 items, e.g., “Taken a photograph of me doing something humiliating and shared it without permission,” α = .90), and personal attack (4 items, e.g., “Called me an offensive nickname,” α = .85). The Cyber Bullying Behaviours scale comprises 12 items across three subscales: sharing images (4 items, e.g., “Made a video of someone doing something humiliating and shared it without permission,” α = .85), gossip (5 items, e.g., “Forwarded a post with a rumour about someone,” α = .85), and personal attack (3 items, e.g., “Made fun of someone because of their appearance,” α = .86). Before completing the scales, participants were told that they should respond to the questions for all forms of electronic forms of contact. Electronic forms of contact were defined as “all types of technology that may be used to communicate with others.” The factor structure of the scales has been previously established and confirmed, and convergent validity is similar to other cyber bullying scales (Çetin et al. 2011) for involvement in face-to-face bullying and social desirability (Betts and Spenser 2017). The variation in the number of items reflects the psychometric properties of the scale that is reported elsewhere (Betts and Spenser 2017).

For both scales, participants responded using a 6- point scale ranging from 1 (*Never*) to 6 (*Everyday*). Responses from the Cyber Victimisation Experiences scale and Cyber Bullying Behaviours scale were used to indicate participants’ experiences of cyber bullying as a (a) victim (derived from the average response to the cyber victimisation experiences scale), (b) bully (derived from the average response to the cyber bullying behaviours scale), and (c) bully/victim (derived from the average response to all items in the cyber victimisation experiences scale and cyber bullying behaviours scale). Higher scores indicated greater endorsement of each cyber bullying role.

#### Self-Esteem

Harter’s (1982) 7-item general self-worth scale from the Perceived Competence scale was used to assess participants’ self-esteem (e.g., “I am sure of myself”) on a 5-point scale ranging from 1 (*strongly disagree*) to 5 (*strongly agree*). Items were coded and summed such that high scores indicated higher self-esteem, and the scale demonstrated good internal consistency reliability (α = .84) that was comparable to previous studies (Butler and Gasson 2005).

#### Trust

Five items from Flanagan and Stout’s (2010) social trust (e.g., “In general, most people can be trusted”) and interpersonal trust (e.g., “I have friends that I can trust to keep a secret”) measures were used to assess trust. Participants reported the extent to which they agreed with the trust items using a 5-point scale ranging from 1 (*strongly disagree*) to 5 (*strongly agree*). Items were summed such that higher scores indicated stronger trust. Given the small number of items in the scale, there was modest internal consistency (α = .60).

#### Perceived Peer Acceptance

The 13-item peer acceptance subscale form the Coping Resources Inventory Scale for Educational Enhancement (McCarthy et al. 2000) assessed self-reported perceived peer acceptance (e.g., “Classmates are good to me”). Participants responded to the items using a 5-point scale ranging from 1 (*strongly disagree*) to 5 (*strongly agree*), and responses were coded and summed such that higher scores indicated higher peer acceptance. The scale demonstrated good internal consistency reliability (α = .84), similar to the internal consistency reliability in the original development of the scale (α = .80; McCarthy et al. 2000).

#### Perceptions of the Value of Learning and the Importance of School

The 33-item Secondary Learners Survey (Underwood et al. 2010) was used to assess participants’ perceptions of the value of learning and the importance of school. The Secondary Learners Survey assesses young people’s experiences of school and their associated attitudes across domains of learning (e.g., “The teachers in this school understand and support me”), lessons (e.g., “My teacher helps me to understand my own way of learning”), doing well (e.g., “I expect to so well in school this year”), challenge (e.g., “Once I have solved a problem my teacher gives me a harder task”), value (e.g., “It is important to me to do well in school”), persistence (e.g., “I work hard to get good marks even when I don’t like the topic”), and taking part (e.g., “I don’t really care about school anymore”). Participants recorded their responses using a 6-point scale ranging from 1 (*strongly agree*) to 6 (*strongly disagree*), and items (after recoding as needed) were summed such that higher scores indicated greater perceptions of learning and school. The scale demonstrated very good internal consistency reliability (α = .90).

## Results

### Analysis Overview

To test Hypothesis 1 that involvement in cyber bullying would directly predict perceptions of learning and school and Hypothesis 2 that gender would moderate the relationship between cyber bullying involvement as a victim, bully, or bully/victim and negative perceptions of learning and school, via self-esteem, trust, and perceived peer-acceptance, we used conditional process analysis (Hayes 2013). Conditional process analysis examines both conditional effects (i.e., gender) and potential indirect effects (i.e., trust, self-esteem, and perceived peer acceptance). The mediation (indirect) effects were tested using bias-corrected bootstrapping with 10,000 sample draws. The 95% confidence intervals are reported. This mediated moderation technique has been used previously to examine the mediating effect of resilience and the moderating effect of mindfulness in the relationship between bullying victimisation and depressive symptoms (Zhou et al. 2017). In the present research, we used the SPSS macro PROCESS (Hayes 2013) to undertake the analysis. Table 1 presents the descriptive statistics for the variables of interest for each gender. The variables used to assess cyber bullying involvement as a victim, bully, and bully/victim were treated as continuous to overcome issues associated with creating cut-off scores that can alter the proportion of individuals classified as a bully, victim, bully/victim depending on the criteria used (see Betts et al. 2016).

**Table 1:** Descriptive statistics for study variables by participants’ gender

	Young Men		Young Women	
	*M*	*SD*	*M*	*SD*
Cyber bullying involvement as a victim	1.50	.82	1.44	.55
Cyber bullying involvement as a bully	1.36	.64	1.25	.37
Cyber bullying involvement as a bully/victim	1.43	.66	1.35	.42
Self-esteem	28.11	4.25	26.46	4.69
Trust	12.66	2.08	13.20	1.67
Perceived peer acceptance	46.87	7.99	46.77	7.40
Perceptions of learning and school	138.29	19.40	139.90	21.51

### Victim, Bully, or Bully/Victim

The overall models for involvement in cyber bullying as a victim and perceptions of learning and school, *R* ^*2*^ = .27, *F*(9,275) = 11.35, *p* < .001, involvement in cyber bullying as a bully and perceptions of learning and school, *R* ^*2*^ = .26, *F*(9,275) = 10.66, *p* < .001, and involvement in cyber bullying as a bully/victim and perceptions of learning and school, *R* ^*2*^ = .28, *F*(9,275) = 11.69, *p* < .001, were significant (see Table 2). In all models, perceived peer acceptance negatively predicted perceptions of learning and school. Also, in all models there was a significant interaction between gender and involvement in cyber bullying as either a victim, bully, or bully/victim (dependent on the model). Finally, there was also a significant interaction in all models between gender and perceived peer acceptance. Together, these results indicate the presence of conditional direct and indirect effects respectively.

**Table 2:** Overall model summaries for the analyses predicting perceptions of learning and school

Variables	β	*SE*	*t*	*p*	95% CI
(a) Cyber bullying involvement as a victim					
Constant	133.69	32.61	4.10	<.001	[69.50, 197.87]
Victim	4.16	4.92	.85	.398	[-5.52, 13.84]
Self-esteem	1.17	1.00	1.17	.244	[-.80, 3.13]
Trust	2.23	1.89	1.18	.239	[-1.49, 5.96]
Perceived peer acceptance	-1.38	.56	-2.47	.014	[-2.48, -.28]
Gender	-19.98	20.41	-.97	.329	[-60.15, 20.20]
Victim x gender	-7.94	3.45	-2.30	.022	[-14.73, -1.16]
Self-esteem x gender	-.34	.61	-.57	.572	[-1.54, .85]
Trust x gender	-1.18	1.26	-.94	.348	[-3.65, 1.29]
Perceived peer acceptance x gender	1.25	.36	3.47	.006	[.54, 1.95]
(b) Cyber bullying involvement as a bully					
Constant	131.14	31.64	4.15	<.001	[68.86, 193.42]
Bully	5.53	6.51	.85	.396	[-7.28, 18.33]
Self-esteem	1.18	1.01	1.17	.241	[-.80, 3.17]
Trust	2.43	1.91	1.28	.204	[-1.32, 6.18]
Perceived peer acceptance	-1.40	.57	-2.47	.014	[-2.51, -.28]
Gender	-21.24	19.99	-1.06	.289	[-60.59, 18.12]
Bully x gender	-10.02	4.75	-2.12	.036	[-19.36, -.67]
Self-esteem x gender	-.35	.61	-.57	.567	[-1.56, .86]
Trust x gender	-1.33	1.26	-1.05	.292	[-3.82, 1.16]
Perceived peer acceptance x gender	1.34	.36	3.71	<.001	[.63, 2.05]
(c) Cyber bullying involvement as a bully/victim					
Constant	130.17	32.38	4.019	<.001	[66.41, 193.92]
Bully/victim	6.06	6.10	.99	.322	[-5.95, 18.07]
Self-esteem	1.17	1.00	1.17	.243	[-.80, 3.13]
Trust	2.23	1.88	1.18	.238	[-1.48, 5.94]
Perceived peer acceptance	-1.34	.56	-2.41	.017	[-2.43, -.24]
Gender	-16.28	20.44	-.80	.426	[-56.52, 23.95]
Bully/victim x gender	-10.98	4.36	-2.52	.012	[-19.58, -2.40]
Self-esteem x gender	-.37	.61	-.61	.541	[-1.57, .83]
Trust x gender	-1.17	1.25	-.93	.352	[-3.62, 1.30]
Perceived peer acceptance x gender	1.25	.36	3.49	<.001	[.54, 1.95]

#### Conditional Direct Effects

Table 3 outlines the conditional direct effects. There was a significant negative conditional direct effect of involvement in cyber bullying as a victim, bully, or bully/victim and perceptions of learning and school for female adolescents. Experiencing higher levels of cyber bullying as a victim predicted lower perceptions of learning and school for young women. Similarly, engaging in higher levels of cyber bullying as a bully predicted lower perceptions of learning and school for young women. Also, fulfilling the role of bully/victim in cyber bullying predicted lower perceptions of learning and school for young women. For young men, the only significant conditional direct effect was between involvement in cyber bullying as a bully/victim and perceptions of learning and school. Experiencing and engaging in higher levels of cyber bullying negatively predicted lower perceptions of learning and school.

**Table 3:** Conditional direct effects for cyber bullying involvement and perceptions of learning and school by students’ gender

Students	β	*SE*	*t*	*p*	95% CI
(a) Cyber bullying involvement as a victim					
Young Men	-3.78	2.02	-1.87	.063	[-.52, .01]
Young Women	-11.73	2.79	-4.20	<.001	[-1.15, -.42]
(b) Cyber bullying involvement as a bully					
Young Men	-4.49	2.57	-1.75	.081	[-9.55, .56]
Young Women	-14.51	3.99	-3.63	<.001	[-22.37, -6.65]
(c) Cyber bullying involvement as a bully/victim					
Young Men	-4.93	2.46	-2.00	<.05	[-9.78, -.07]
Young Women	-15.92	3.60	-4.42	<.001	[-1.86, -.55]

#### Conditional Indirect Effects

Table 4 reports the conditional indirect effects and provides partial support for Hypothesis 2. Perceived peer acceptance mediated the relationship between involvement in cyber bullying as a (a) victim and perceptions of learning and school, (b) bully and perceptions of learning and school, and (c) bully/victim and perceptions of learning and school for young women. Experiencing higher levels of cyber bullying as a victim predicted lower perceived peer acceptance which, in turn, predicted lower perceptions of learning and school. Similarly, engaging in higher levels of cyber bullying as a bully predicted lower perceived peer acceptance which, in turn, predicted lower perceptions of learning and school. Also, experiencing higher levels of cyber bullying as a victim and engaging in higher levels of cyber bullying behaviours as a bully predicted lower perceived peer acceptance which, in turn, predicted lower perceived peer acceptance. The comparable relationships were not significant for young men, and gender differences in these relationships were significant according to the index of moderated mediation (Table 5).

**Table 4:** Conditional indirect effects of trust, self-esteem, and perceived peer acceptance for cyber bullying involvement and perceptions of learning and school by students’ gender

Mediator	Gender	β	Boot *SE*	Boot 95% CI
(a) Cyber bullying involvement as a victim				
Trust	Male	-.28	.44	[-1.79, .16]
	Female	.03	.36	[-.48, 1.15]
Self-esteem	Male	-.99	.83	[-3.02, .27]
	Female	-1.26	1.67	[-5.07, 1.53]
Perceived peer acceptance	Male	.19	.49	[-.57, 1.51]
	Female	-5.09	2.10	[-10.49, -1.88]
(b) Cyber bullying involvement as a bully				
Trust	Male	-.04	.45	[-1.37, .62]
	Female	.04	.37	[-.42, 1.35]
Self-esteem	Male	-.63	.68	[-2.79, .17]
	Female	-1.41	2.01	[-6.16, 1.93]
Perceived peer acceptance	Male	-.05	.43	[-1.51, .50]
	Female	-4.79	.265	[-12.28, -1.28]
(c) Cyber bullying involvement as a bully/victim				
Trust	Male	-.23	.49	[-1.99, .26]
	Female	.03	.43	[-.61, 1.38]
Self-esteem	Male	-1.01	.85	[-3.31, .23]
	Female	-1.45	2.16	[-6.32, 2.23]
Perceived peer acceptance	Male	.07	.35	[-.39, 1.22]
	Female	-6.20	2.64	[-13.03, -2.22]

**Table 5:** Index of moderated mediation

Mediator	Index	Boot *SE*	95% CI
(a) Cyber bullying involvement as a victim			
Trust	.32	.56	[-4.28, 3.12]
Self-esteem	-.27	1.86	[-4.28, 3.12]
Perceived peer acceptance	-5.28	2.16	[-10.67, -1.93]
(b) Cyber bullying involvement as a bully			
Trust	.08	.59	[-.89, 1.60]
Self-esteem	-.78	2.11	[-5.42, 2.96]
Perceived peer acceptance	-4.74	2.68	[-12.18, -1.16]
(c) Cyber bullying involvement as a bully/victim			
Trust	.26	.66	[-.69, 2.10]
Self-esteem	-.44	2.32	[-5.24, 3.87]
Perceived peer acceptance	-6.26	2.67	[13.23, -2.27]

Therefore, there was partial support for Hypothesis 2 because perceived peer acceptance mediated the relationship between involvement in cyber bullying as a victim, bully, or bully/victim and perceptions of learning and school in young women. However, trust and self-esteem did not mediate the relationship between involvement in cyber bullying and perceptions of learning and school.

## Discussion

In the current study we examined the extent to which the predominately out-of-school experience of cyber bullying impacted young people’s perceptions of school. Our results revealed evidence that gender moderated the relationship between involvement in cyber bullying and perceptions of learning and school. For young women, the main findings of the study were that (a) a relationship occurred between involvement in cyber bullying as a victim, bully, or bully/victim and (b) the relationship between involvement in cyber bullying as a victim, bully, or bully/victim and perception of learning and school was mediated by perceived peer acceptance. For young men, the only significant relationship was between involvement in cyber bullying as a bully/victim and perceptions of learning and school. Self-esteem and trust failed to mediate the relationship between involvement in cyber bullying and perceptions of learning and school for both young women and young men.

A relationship occurred between involvement in cyber bullying as a victim, bully, or bully/victim and perceptions of learning and school for young women: Young women who were more involved in cyber bullying as a victim, bully, or a bully/victim had more negative perceptions of learning and school. Consequently, there is evidence that, for young women, their experiences of cyber bullying, which typically occur outside the school environment, impact their perceptions of learning and school. This finding suggests that factors outside school can spill over to negatively impact young women’s views of learning and the value of school. Although similar spillover has been documented between family stressors and the school environment for young people (Timmons and Margolin 2015), and such spillover is predictive of poorer academic performance (Flook and Fuligni 2008), few studies have examined involvement in cyber bullying as both a victim and bully simultaneously. Previous research has reported that young people who experience cyber bullying are more likely to avoid school (West 2015), be fearful of school (Raskauskas and Stoltz 2007), and have negative attitudes toward school (Bayar and Ucanok 2012; Pyzalski 2012). Consequently, it seems that involvement in cyber bullying not only impacts negative attitudes towards school (as identified in previous research), but also impacts young women’s attitudes toward learning.

For young women, the relationship between cyber bullying involvement and perceptions of learning and school was mediated by perceived peer acceptance such that involvement in cyber bullying as a victim, bully, or a bully/victim predicted lower perceived peer acceptance and perceived peer acceptance, in turn, predicted positive perceptions of learning and school. This finding contributes to the growing evidence base that involvement in cyber bullying undermines young people’s peer relationships (Jackson and Cohen 2012; Vandebosch and van Cleemput 2009) and underscores the importance of peer relationships for fostering positive attitudes toward learning and school (Lynch et al. 2013). Further, the nature of the mediation relationship provides evidence that positive perceptions of peer relationships can act as a protective buffer for young women against the effects of cyber bullying similar to the friendship protection hypothesis advanced for face-to-face bullying (Kendrick et al. 2012).

For young men, our study revealed a significant relationship only between cyber bullying involvement as a bully/victim and perceptions of learning and school; no other relationships were significant. There are two possible explanations for this pattern. First, males report that cyber bullying has less of an impact on them than it does for females. For example, over half of males who experience cyber bullying report they are “only a little bit upset” and that it is less upsetting than face-to-face bullying (Sakellariou et al. 2012). Similarly, Bauman and Newman (2013) found that males reported less distress than females did when they experienced cyber bullying. This comparably lower level of distress attributed to cyber bullying may mean that the effects of cyber bullying involvement are less likely to spill over into school for young men who are exclusively victims and bullies. However, fulfilling the bully/victim role may result in a cumulative impact on adjustment (Wolke et al. 2013), and males are more likely to fulfil this role than are females (Yang and Salmivalli 2013), resulting in the current findings.

Second, the age of our sample may go some way to explain the pattern of (non)findings. A recent meta-analysis of 122 effect size estimates revealed that females were more likely to report cyber bullying during early-to-mid adolescence whereas males reported higher levels of cyber bullying involvement during late adolescence (Barlett and Coyne 2014). Barlett and Coyne (2014) account for the pattern of findings by pointing to females’ more sophisticated knowledge of the social structure during early adolescence and by arguing that females’ initial involvement in cyber bullying reflects their comparatively earlier maturation, but then males “catch up” by late adolescence.

The current findings may also reflect gender differences in how young people engage with digital technology. Although females may be more likely to use digital technology to maintain friendships and gain self-esteem from doing so (Barker 2009), the benefits for males communicating via digital technology with their friends are higher than they are for females (Valkenburg and Peter 2009). Given the spillover we identified for young women who fulfil all cyber bullying roles and for young men who are bully/victims, tackling involvement in cyber bullying may go some way to ameliorate its negative effects.

### Limitations and Future Research Directions

There are a number of limitations of the current study. First, the cross-sectional nature of the study means that causality cannot be established in the relationship between young peoples’ involvement in cyber bullying and perceptions of learning and school. Therefore, future research should adopt longitudinal methods to further determine causality in the relationship between cyber bullying involvement and school adjustment. Longitudinal designs and comparative designs could also be used to examine the developmental trajectories of cyber bullying involvement for learning to explore whether similar findings emerge across the various educational stages (i.e., elementary, high school, college). Second, as with other studies assessing cyber bullying, the current study used self-report methods. According to Runions (2013), future research should consider using experience sampling methods to determine young people’s involvement in cyber bullying to overcome issues around potential under-reporting. There is evidence that young people often under-estimate their involvement in cyber bullying because they are afraid that their access to technology will be removed (Mishna et al. 2009). However, it should be noted in the current study that it was made clear to young people that their answers would remain confidential (unless they disclosed very high and significant involvement in cyber bullying). Third, participants who completed the questionnaires were significantly older than those who did not complete the questionnaires. However, the demographics of the final sample still reflected the age group of participants most likely to be at risk of cyber bullying (Kowalski and Limber 2007; Ortega et al. 2009).

### Practice Implications

The findings of the current paper have two main practice implications. First, they highlight that, for young women, experiences outside school impact their perceptions of learning and school. Specifically, involvement in cyber bullying as a victim, bully, or bully/victim adversely impacted young women’s attitudes toward school. Together, these findings contribute to the growing body of evidence that highlights how stressors outside the school environment can spill over to young women’s schooling (Timmons and Margolin 2015). Therefore, educational practitioners need to be mindful that young women who are involved in cyber bullying may not be in a position to take advantage of the educational opportunities afforded to them. Consequently, understanding the nature of the spillover for young women involved in cyber bullying for their perceptions of school will enable practitioners to support young people so that the potential adverse impact of the stressors are lessened.

Second, based on the findings of the current study, it seems appropriate to consider interventions that reduce cyber bullying that focus on strengthening and improving social networks, especially for young women. For young women, the role of perceived peer acceptance as a mediator in the relationship between cyber bullying involvement as a victim, bully, or bully/victim and perceptions of learning and school suggests that key to buffering young women from the adverse effects of cyber bullying is to develop their social relationships. One mechanism through which this could occur would be to further enhance peer-support interventions similar to those proposed by Huston and Cowie (2007). Huston and Cowie (2007) developed and evaluated a digital peer support scheme that allowed young people to anonymously seek support from their peers, enabling them to overcome the potential barriers of other preconceptions and personalities. In other words, fostering positive peer relationships may be a protective factor for young women who experience cyber bullying. However, it is important to recognise that young people involved in cyber bullying may develop cognitive biases aligned to suspicion and reluctance to interact which may adversely influence social relationships. Further, as Thomas et al. (2016) note, young female and male students require different versions of anti-bullying interventions because of gender differences in how they internalise and express wrongdoing in the digital world. Therefore, interventions designed to address such biases and gender differences are required.

### Conclusions

For young women, involvement in cyber bullying negatively predicted perceptions of learning and school, and this relationship was mediated by perceived peer acceptance. Therefore, it seems for young women that their experiences of cyber bullying, regardless of whether they fulfil the victim, bully, of bully/victim role in cyber bullying, traverse into the school environment by negatively impacting their perceptions of learning and school. Further, the current study highlighted the importance of perceived peer acceptance as a protective factor for young women. For young men, fulfilling the bully/victim role negatively impacted their perceptions of learning and school. Consequently, enhancing young women’s social relationship quality and understanding the unique cumulative effect of being a bully/victim for young men would likely help to protect them from the negative effects of involvement in cyber bullying. The current findings have implications for practitioners working with young people who are involved in cyber bullying because this external stressor clearly has negative implications for their attitudes toward school and the value they place on learning.
